# Identifying Depression Subtypes and Investigating their Consistency and Transitions in a 1-Year Cohort Analysis

**DOI:** 10.1192/j.eurpsy.2024.483

**Published:** 2024-08-27

**Authors:** C. Oetzmann, N. Cummins, F. Lamers, F. Matcham, K. M. White, J. M. Haro, S. Siddi, S. Vairavan, B. W. Penninx, V. A. Narayan, M. Hotopf, E. Carr

**Affiliations:** ^1^Psychological Medicine; ^2^Biostatistics & Health Informatics, King’s College London, London, United Kingdom; ^3^Department of Psychiatry, Amsterdam UMC, location Vrije Universiteit; ^4^Mental Health Program, Amsterdam Public Health, Amsterdam, Netherlands; ^5^School of Psychology, University of Sussex, Falmer, United Kingdom; ^6^Parc Sanitari Sant Joan de Déu, Fundació Sant Joan de Déu, CIBERSAM, Universitat de Barcelona, Barcelona, Spain; ^7^Research and Development, Janssen, Titusville, United States; ^8^Davos Alzheimer’s collaborative, Geneva, Switzerland

## Abstract

**Introduction:**

Major Depressive Disorder (MDD) is a complex mental health condition characterized by a wide spectrum of symptoms. According to the Diagnostic Statistical Manual 5 (DSM-5) criteria, patients can present with up to 1,497 different symptom combinations, yet all receive the same MDD diagnosis. This diversity in symptom presentation poses a significant challenge to understanding the disorder in the wider population. Subtyping offers a way to unpick this phenotypic diversity and enable improved characterization of the disorder. According to reviews, MDD subtyping work to date has lacked consistency in results due to inadequate statistics, non-transparent reporting, or inappropriate sample choice. By addressing these limitations, the current study aims to extend past phenotypic subtyping studies in MDD.

**Objectives:**

(1) To investigate phenotypic subtypes at baseline in a sample of people with MDD;

(2) To determine if subtypes are consistent between baseline 6- and 12-month follow-ups; and

(3) To examine how participants move between subtypes over time.

**Methods:**

This was a secondary analysis of a one-year longitudinal observational cohort study. We collected data from individuals with a history of recurrent MDD in the United Kingdom, the Netherlands and Spain (N=619). The presence or absence of symptoms was tracked at three-month intervals through the Inventory of Depressive Symptomatology: Self-Report (IDS-SR) assessment. We used latent class and three-step latent transition analysis to identify subtypes at baseline, determined their consistency at 6- and 12-month follow-ups, and examined participants’ transitions over time.

**Results:**

We identified a 4-class solution based on model fit and interpretability, including (Class 1) severe with appetite *increase*, (Class 2), severe with appetite *decrease*, (Class 3) moderate, and (Class 4) low severity. The classes mainly differed in terms of severity (the varying likelihood of symptom endorsement) and, for the two more severe classes, the type of neurovegetative symptoms reported (Figure 1). The four classes were stable over time (measurement invariant) and participants tended to remain in the same class over baseline and follow-up (Figure 2).

**Image:**

**Figure:**
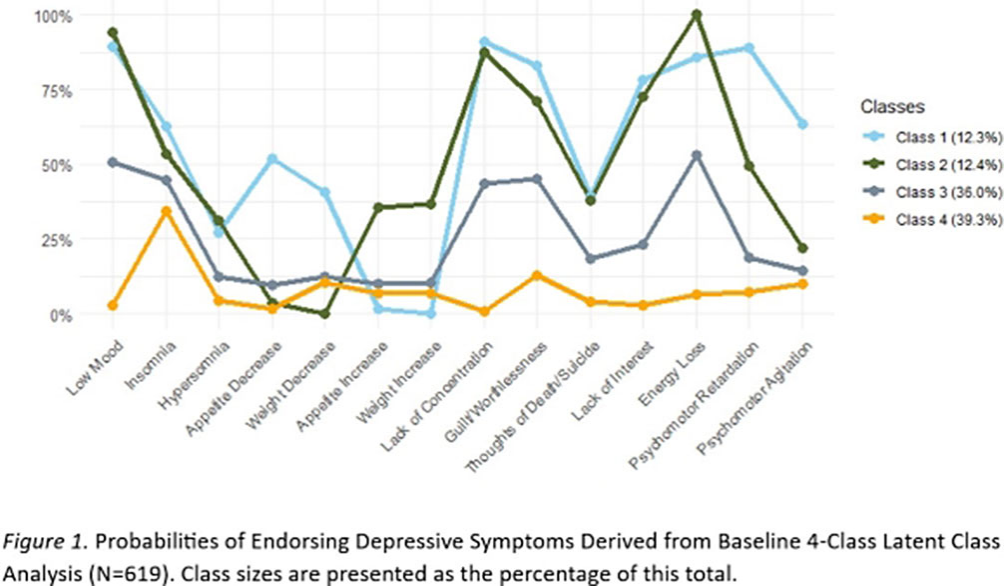

**Image 2:**

**Figure:**
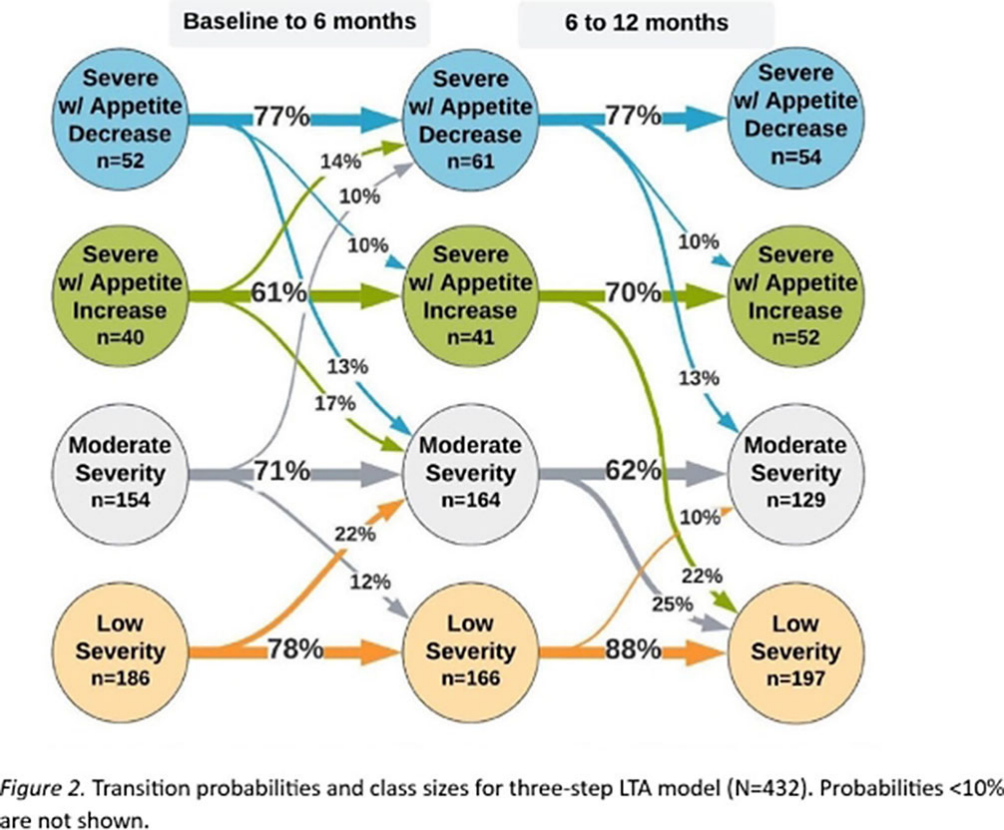

**Conclusions:**

We identified four stable subtypes of depression, with individuals most likely to remain in their same class over 1-year follow-up. This suggests a chronic nature of depression, with (for example) individuals in severe classes more likely to remain in the same class throughout follow-up. Despite the vast heterogeneous symptom combinations possible in MDD, our results emphasize differences across severity rather than symptom type. This raises questions about the meaningfulness of these subtypes beyond established measures of depression severity. Implications of these findings and recommendations for future research are made.

**Disclosure of Interest:**

C. Oetzmann Grant / Research support from: C.O. is supported by the UK Medical Research Council (MR/N013700/1) and King’s College London member of the MRC Doctoral Training Partnership in Biomedical Sciences., N. Cummins: None Declared, F. Lamers: None Declared, F. Matcham: None Declared, K. White: None Declared, J. Haro: None Declared, S. Siddi: None Declared, S. Vairavan Employee of: S.V is an employee of Janssen Research & Development, LLC and hold company stocks/stock options., B. Penninx : None Declared, V. Narayan: None Declared, M. Hotopf Grant / Research support from: M.H. is the principal investigator of the RADAR-CNS programme, a precompetitive public–private partnership funded by the Innovative Medicines Initiative and the European Federation of Pharmaceutical Industries and Associations. The programme received support from Janssen, Biogen, MSD, UCB and Lundbeck., E. Carr: None Declared

